# Transcultural adaptation and validation of the Persian version of the breast cancer awareness measure (BCAM) questionnaire

**DOI:** 10.1002/cam4.1740

**Published:** 2018-08-27

**Authors:** Zahra Heidari, Awat Feizi

**Affiliations:** ^1^ Endocrine and Metabolism Research Center and Department of Biostatistics and Epidemiology School of Health Isfahan University of Medical Sciences Isfahan Iran

**Keywords:** awareness, breast cancer, instrument development, psychometrics properties, validation studies

## Abstract

**Background/objective:**

Enhancing awareness level about breast cancer is a pivotal strategy for reducing breast cancer burden. There is no fully validated Persian instrument for evaluating breast cancer awareness. This study aimed at investigating the validity and reliability of the Persian version of the Breast Cancer Awareness Measure (BCAM) questionnaire.

**Methods:**

This methodological cross‐sectional study was conducted among 1078 Persian language women (including 965 general women and 113 medical/clinical experts), which selected from different parts of Isfahan city using multistage cluster random sampling method. Translation of BCAM questionnaire was performed using forward‐backward method. Internal consistency was evaluated through Cronbach's *α* and test‐retest reliability using unweighted kappa statistic and intraclass correlation (ICC) coefficient. Construct validity was investigated using both exploratory and confirmatory factor analyses as well as Latent class analysis (LCA), and discriminant validity using ROC curve. Convergent validity was assessed using phi and eta correlation coefficients. Ceiling and floor effects, SE of measurement (SEM), and smallest detectable change (SDC) were also determined.

**Results:**

Persian version of BCAM showed excellent test‐retest reliability (ICC = 0.841) and internal consistency (Cronbach's alpha = 0.882). Most of the computed kappa coefficients were in the range moderate to very good (0.47‐0.81). Medical/clinical experts had higher levels of breast cancer awareness than general women, indicating good discriminant validity (Area under the curve [AUC]) of 0.822 (95% CI: 0.781, 0.864). Construct validity evaluation by exploratory factor analysis (EFA) led to extraction of two factors from 11 items (“breast shape changes” and “breast pain and lump”), and the confirmatory factor analysis (CFA) confirmed the adequacy of extracted construct from EFA. Latent class analysis for evaluating of construct validity led to extracting three classes from participants (high [12.83%], moderate [60.97%], and low [26.2%]) in terms of awareness levels about early warning signs of breast cancer. All item‐scale correlation coefficients exceeded the set value of 0.40, indicating satisfactory convergent validity. No ceiling and floor effects were detected. SEM and SDC were found to be 0.85 and 2.36, respectively.

**Conclusions:**

The Persian version of BCAM is a reliable and valid instrument for monitoring levels of breast cancer awareness in general women population, also it can be used for evaluating the impacts of interventions attempting to raise breast cancer awareness.

## 1 INTRODUCTION

Breast cancer (BC) is the most commonly diagnosed cancer among women, impacting over 1.5 million women each year, and also the second leading cause of cancer‐related deaths in women throughout the world.^1^, ^2^ In Iran also, this disease accounts for 24.4% of all cancers and its incidence has been estimated to be 17.81%, which has increased dramatically in recent years.^3^ Unfortunately, because of resource and infrastructure constraints and lacks of diagnosing at earlier stages in low‐income countries, there is an enormous difference in breast cancer survival rates worldwide, with an estimated 5‐year survival of 80% in developed countries to lower than 40% in developing countries.^4^, ^5^

Delayed diagnosis in developing countries is related, partly, to poor awareness, especially about early warning signs and symptoms of BC.^6^, ^7^ Increasing awareness about breast cancer is widely accepted first line in the battle against BC, especially in those countries lacking ongoing organized population‐based screening program.^8^, ^9^ Previous studies have shown that the increased women's awareness about early diagnosis can change people's screening health‐seeking behaviors.^6^, ^10^, ^11^ Therefore, there is an urgent need for improving great BC awareness and its early detection measures among women.^12^ Accordingly, developing and validating of instruments for measuring and monitoring levels of breast cancer awareness and its determinants could be a pivotal approach.^7^, ^9^ Such standardized instruments can help policy makers to design and evaluate the interventions in order to promote breast cancer awareness efficiently.^7^, ^9^ Although in recent years, several breast cancer awareness instruments have been developed and validated over the world.^7^, ^8^, ^9^, ^12^, ^13^, ^14^, ^15^, ^16^, ^17^, ^18^, ^19^, ^20^, ^21^, ^22^, ^23^ Some of these instruments contain design and/or methodological limitations and are not fully validated.^13^, ^14^, ^15^, ^16^, ^17^ However, United Kingdom Cancer Research center developed a fully validated instrument, that is, Breast Cancer Awareness Measure (BCAM) in 2010.^7^ It is a self‐completed questionnaire for assessing knowledge of breast cancer symptoms and age‐related risk, and also report frequency of breast checking.^7^ In UK population, the readability of the BCAM was reported high and construct validity was supported by significant differences between the levels of breast cancer awareness among cancer experts and general women.^7^, ^18^ However, the BCAM was developed and validated for western populations where etiologic factors and health policy of breast cancer are different considerably from developing nations.^9^ In addition, cross‐cultural and language differences routinely introduce measurement biases which affect the quality of collected data in other populations.^18^, ^19^

In a developing community such as Iran where the late presentation is predominant and majority of breast cancer patients are diagnosed at advanced stages of disease,^6^ there is an urgent need for developing and validating instruments to assess breast cancer awareness. To the best of our knowledge, there is no fully validated instrument for assessing breast cancer awareness in Iran. The current study aimed at developing a Persian version of BCAM questionnaire, according to the guidelines for cross‐cultural adaptation,^20^ and evaluating its psychometric properties (test‐retest reliability, internal consistency, convergent validity, discriminant validity, and construct validity).

## 2 METHODS AND MATERIALS

### 2.1 Study design and participants

This methodological cross‐sectional study was conducted between July 2016 and November 2017 among 1078 Persian women (including 113 medical/clinical experts and 965 general women) in Isfahan, a largest city in central Iran. All medical/clinical experts were randomly selected among female physicians/nurses/midwives from three major hospitals affiliated with Isfahan University of Medical Sciences (“Al‐Zahra,” “Noor and Hazrat‐e‐Ali Asghar,” and “Shahid Beheshti”). General women were selected from different parts of Isfahan city (such as shopping centers, recreational places, kindergartens, health centers, and different streets of the city) using multistage cluster random sampling method. Eligibility criteria for participating in this study were as at least 18 years old, able to read and write Persian, and should be permanent resident of Isfahan. Those participants who did not answer to main questions (items of BCAM questionnaire regarding breast cancer awareness measures) were excluded. Eligible women were invited to participate in the study by face‐to‐face invitation. Interviews were performed by trained interviewers. After obtaining oral consent to participate in the study, we requested from participants to complete the BCAM questionnaire. The study was approved by the bioethics committee of the Isfahan University of Medical Sciences (Project Number: 194126).

### 2.2 The breast cancer awareness measure

UK Cancer Research center developed and validated an awareness measure specific to breast cancer awareness which called BCAM.^7^ It includes items on knowledge of breast cancer symptoms, knowledge of age‐related risk, and reported frequency of breast checking. In UK population, the readability of the BCAM was reported high and over 90% of women found it acceptable. The analyses of test‐retest reliability of the BCAM showed moderate to good reliability for most items. Good construct validity was approved by significant differences between the levels of breast cancer awareness among cancer experts compared to general women (50% vs 6%, *P* = 0.001).^7^, ^18^

### 2.3 Translation and cross‐cultural adaptation

In accordance with the guidelines recommended by Beaton et al,^20^ the “forward‐backward” procedure was applied to translate the BCAM questionnaire from English into Persian (Iranian language), after seeking permission from the initial developer (Louise Linsell, Kings College London, London, UK). First, two professional translators translated the original English instrument into Persian, independently (forward translation). They were native Persian speakers but fluent in both languages. One of the translators was an expert in translation of medical texts, so she was aware of the concept of the questionnaire being translated. However, the second translator was unaware of the concepts being examined in the original English instrument. Then, the current study's researchers (A.F. and Z.H.) and both translators compared the both translated versions with the original questionnaire to develop an acceptable forward translation. One common translation of the English version of BCAM was adopted. Then a bilingual translator, who had not seen the questionnaire previously and lacks of medical background translated the Persian adapted version back into English. The translated English version was compared with the original one with respect to conceptual equivalence by researchers (A.F. and Z.H.). Finally, after a careful review and cultural adaptation process, necessary changes were made, and the provisional Persian version of the BCAM questionnaire was provided. This prefinal Persian BCAM questionnaire was piloted on 50 women aged more than 18 years who volunteered to participate in the study. They were asked to express any difficulty to understand any word or sentences in the questionnaire (face validity). According to the participants’ feedbacks, the translation quality simplicity, and clarity of the questions were verified by most pilot study volunteers. Then, the researchers made final adjustments in response to these feedbacks, and the final Persian version of BCAM was developed. In the process of translation and cross‐cultural adaptation, the following changes were made: for informing the participants that they can choose more than one item from 11 breast cancer early warning signs, we added a sentence as “more than one item is also selectable.” We also added “indentation of nipple or shifting to one side” as an explanation regarding the first item (change in the position of your nipple) for more clarity. We considered “which one of the following woman is more likely to get breast cancer in the next year?” instead of “in the next year, who is most likely to get breast cancer?” question. Besides of demographic characteristics (including age, educational level, marital status, and job), “personal history of breast problems” and “family history of breast cancer,” a further question about the “sources of gaining awareness about breast cancer” was added to the developed questionnaire.

### 2.4 Statistical analysis

In this study, psychometric characteristics of the Persian version of BCAM, including reliability (test‐retest reliability and internal consistency) and validity (construct validity, discriminant validity and convergent validity), were evaluated.

### 2.5 Reliability

We investigated two aspects of reliability, that is, test‐retest reliability and internal consistency. In order to test the extent to which the measure was repeatable (the stability of the measurement over time), we recruited 50 women aged 18 years old and over. The women were asked to complete the BCAM questionnaire at two separate days with a 7‐day interval. Test‐retest reliability was assessed separately for each item using the unweighted kappa statistic (<0.20: poor, 0.21‐0.40: fair, 0.41‐0.60: moderate, 0.61‐0.80: good, 0.81‐1.00: very good).^21^ In addition, intraclass correlation (ICC) coefficient using two‐way mixed model, along with 95% confidence was used to evaluate the relative reliability for the total score of items. ICC ≥0.70 was considered as the evidence of excellent stability.^22^

Internal consistency was evaluated using Cronbach's *α* coefficient (>0.7: acceptable, >0.8: good, and >0.9: excellent).^22^ Data collected in the first administration of the BCAM questionnaire were used to evaluate internal consistency.

### 2.6 Validity

#### 2.6.1 Construct validity

The factor structure of the BCAM was explored using the exploratory factor analysis (EFA). Factor‐item loadings values greater than 0.40 were considered as satisfactory for assigning an item to a specific factor. The data viability for factorability was guided through Kaiser‐Meyer‐Olkin (KMO) measure of sample adequacy (Values > 0.7) and Bartlett's test of Sphericity (*P* < 0.05).^23^ We found two interpretable factors based on the loaded items in each factor. Then, we conducted a confirmatory factor analysis (CFA) in order to assess how well the factors extracted from EFA fit to the observed data. We used four fit indices (chi‐square/*df* [relative chi‐square], root mean square error of approximation [RMSEA], comparative fit index [CFI], and Tucker‐Lewis index [TLI]) for evaluating the goodness of fit model. Relative chi‐square <5.00, a CFI and TLI value of >0.90, and a RMSEA value of <0.08 were considered as acceptable model fit.^23^, ^24^

The latent structure of the BCAM was also investigated using latent class analysis (LCA). In other words, the level of “awareness” about breast cancer warning signs was considered as a latent construct and it was evaluated using LCA. LCA is a parallel approach or counterpart, with factor analysis, but it applicable for categorical variables.^25^ LCA similar to factor analysis addresses the complex patterns of associations that appears among observations; however, unlike factor analysis, in LCA, the underlying unobserved variables are not continuous (dimensions) but are classes or discrete.^25^ This model examines the pattern of relations among a set of observed categorical variables (here “awareness” about breast cancer warning signs) and classifies similar individuals in terms of awareness levels into homogeneous latent classes. This leads participants within each latent class are highly similar to each other and uniquely different from the other classes across the set of evaluated items. Accordingly, comparisons can be made across latent classes with regard to items evaluated (here breast cancer warning signs). We fitted various LCA models with different latent classes. The adequacy of fitted models was guided through comparing the Bayesian Information Criterion (BIC), the Akaike information criterion (AIC), and entropy indices across models. A model with lower “BIC and AIC” and higher “entropy” values indicates better fitting and class separation, respectively.^25^ Then, we performed a cross‐validation, splitting the sample into two subsamples randomly. LCA models with different latent classes were performed on the first half sample (training sample). The adequacy of fitted models was guided through comparing BIC, AIC, and entropy indices across fitted models. Subsequently, we performed a confirmatory LCA (CLCA) on the other subsample (validation sample) to confirm the obtained new solution derived from LCA on training sample. The cross‐validation process was then repeated, with the second half sample as the training data. Finally, the two obtained results in this process were averaged to produce a single estimate.

#### 2.6.2 Discriminant validity

Discriminant validity was assessed based on the BCAM ability to discriminate between general women and medical/clinical experts in terms of awareness levels about breast cancer warning signs. The validity of the measure is supported if distribution of the BCAM items is significantly different across two groups. We distributed the BCAM questionnaire to 965 general women and 113 medical/clinical experts and compared their responses. We tested difference in the distribution of correct answer to each item and total score of correct answer to all items between two groups using chi‐square test or independent Student's *t* test. In addition, receiver operating characteristic (ROC) curve along with the sensitivity and specificity values was used to gauge the ability of the total score of warning signs subscale of BCAM to discriminate between general women and medical/clinical experts. We also assessed the BCAM ability to discriminate between participants with “personal and family history of breast problems” and general women in terms of awareness levels about breast cancer warning signs using chi‐square test or independent Student's *t* test and ROC curve.

#### 2.6.3 Convergent validity

As both the items and the underlying structure of the BCAM questionnaire (extracted latent classes based on items) were discrete variables, the phi correlation coefficient was used to examine item‐scale correlations, corrected for overlap (ie, the phi coefficient was computed between each item and its own domain [with the item removed]). We also used eta correlation coefficient corrected for overlap to assess correlation between each item and the total score of items (subscale of awareness level about warning signs of BCAM). Item convergent validity should be at least 0.40.^25^

#### 2.6.4 Ceiling or floor effects

Ceiling and floor effects were assessed on the first administration of the BCAM to determine content validity. Ceiling/floor effects were considered to be present if more than 15% of the participants achieved the highest or lowest possible scores.^22^

#### 2.6.5 Other statistical analysis

In this study, quantitative and qualitative variables were expressed as mean (SD) and number (percent), respectively. Additional data about age, education level, marital status, job status, personal history of breast problems, family history of breast cancer, and sources of gaining awareness about breast cancer were also collected. The determinants of the awareness level about breast cancer warning signs were evaluated using “latent class regression” analysis (LCR). In the LCR, covariates were included to describe both the formation of the latent classes and how they may be differently measured by the observed indicators. The prediction of latent class membership is obtained by multinomial regression of latent class variable on covariates. The standard error of measurement (SEM) was measured as $\text{SD}\times \sqrt{1-r}$.^22^ The smallest detectable change (SDC) was calculated as $1.96\times \sqrt{2}\times \text{SEM}$.^22^ Data analyses were performed using SPSS (version 16; SPSS Inc., Chicago, IL, USA) and R free statistical software version 3.2.2.

## 3 RESULTS

### 3.1 Participant characteristics

A total of 1078 women participated in this validation study, including 965 (89.5%) general women and 113 (10.5%) medical/clinical experts. The mean (SD) age was 36.5 (11.65) and 34.28 (7.14) years old for general women and medical/clinical experts, respectively. The majority of the participants were married (73% for both groups). Approximately half (50.2%) of the women had academic level of education, and only 28.5% were employed. Personal history of breast problems was reported by 159 (16.6%) and 12 (10.8%) of general women and medical/clinical experts, respectively. Among women with the breast problems, history of breast cancer was reported by 7/159 and 1/12 of general women and medical/clinical experts, respectively. In addition, family history of breast cancer was reported by 198 (20.9%) and 19 (16.8%) of general women and medical/clinical experts, respectively. (Table 1)

**Table 1:** Participants characteristics by studied groups

		General women (n = 965)	Medical/clinical experts (n = 113)
Age		36.50 (11.65)	34.28 (7.14)
Educational level	Illiterate	32 (3.3)	—
	Under Diploma (<12 y)	158 (16.4)	—
	Diploma (12 y)	273 (28.3)	—
	Collegiate (>12 y)	502 (52.0)	113 (100)
Marital status	Single	195 (20.8)	29 (26.1)
	Married	690 (73.5)	81 (73.0)
	Widow	54 (5.8)	1 (0.9)
Job	Housewife	580 (60.7)	—
	Employee	272 (28.5)	113 (100)
	Student	90 (9.4)	—
	Retired	14 (1.5)	—
Personal history of breast problems	No	800 (83.4)	99 (89.2)
	Yes	159 (16.6)	12 (10.8)
	*Breast cancer*	7 (4.7)	1 (10.0)
	*Breast pain*	68 (45.6)	4 (40.0)
	*Benign sickness*	39 (26.2)	4 (40.0)
	*Other problems*	35 (23.5)	1 (10.0)
Family history of breast cancer	No	751 (79.1)	94 (83.2)
	Yes	198 (20.9)	19 (16.8)
	*Mother*	1 (5.6)	26 (12.7)
	*Sister*	1 (5.6)	25 (12.3)
	*Daughter*	0	2 (1.0)
	*Grand Mother*	1 (5.6)	11 (5.4)
	*Aunt*	3 (16.7)	51 (25.0)
	*Other sources*	12 (66.7)	89 (43.6)

Values are mean (SD) or number (percent).

### 3.2 Reliability analyses

#### 3.2.1 Test‐retest reliability

Test‐retest reliability was evaluated by calculating the kappa and intra‐class correlation (ICC) statistics over a week interval in a subsample of 50 women. Results of the test‐retest reliability of the BCAM are shown in Table 2. Most of the computed kappa coefficients were in the range “moderate” to “very good” (0.47‐0.81); and the percentages of agreement between correct responses to the items at the two times (test and retest stages) were high (73%‐95%) for proposed items).

**Table 2:** Test‐retest reliability of the breast cancer awareness measure

	ICC	95% CI for ICC
The total score of warning signs	0.841	(0.74, 0.91)^a^
	**Kappa statistic (SE)** a	**% of exact agreements**
Knowledge of breast cancer symptoms (yes/no)	0.81 (0.140)	95.92
Know if the following are warning signs (yes/no)		
Change in nipple position	0.72 (0.142)	85.71
Pulling in of nipple	0.64 (0.143)	85.71
Puckering/dimpling	0.47 (0.140)	73.47
Lump in breast	0.63 (0.142)	83.67
Redness of skin	0.60 (0.143)	81.63
Change in size	0.64 (0.140)	81.63
Pain in breast/armpit	0.71 (0.143)	87.76
Discharge from nipple	0.68 (0.142)	85.71
Nipple rash	0.67 (0.141)	85.71
Lump under armpit	0.66 (0.143)	85.71
Change in shape	0.75 (0.142)	87.76
Knowledge of age‐related risk (a 30 y old/50 y old/70 y old/a woman of any age)	0.75 (0.102)	85.71
Breast checking (rarely or never/at least/every 6 mo/once a month/once a week)	0.75 (0.098)	83.67

a All presented Kappa values and ICC are significant at *p*‐value < 0.01

The ICC coefficient for the total score of warning signs subscale suggests strong test‐retest reliability (ICC = 0.841, 95% CI: 0.74 to 0.91, Table 2). The ICC coefficients for the total score of extracted subscales “breast shape changes” and “breast pain and lump” factors (Table 2) were estimated to be 0.81 and 0.85, respectively. The SEM and the SDC for the BCAM‐Persian were computed to be 0.85 (95% CI ± 1.641) and 2.36, respectively.

#### 3.2.2 Internal consistency

The internal consistency of the warning signs subscale of BCAM was evaluated using Cronbach's alpha = 0.882 indicating strong internal reliability. The Cronbach's alpha coefficients for the total score of extracted subscales “breast shape changes” and “breast pain and lump” factors were estimated to be 0.893 and 0.919, respectively.

### 3.3 Discriminant validity

**Figure 1:**
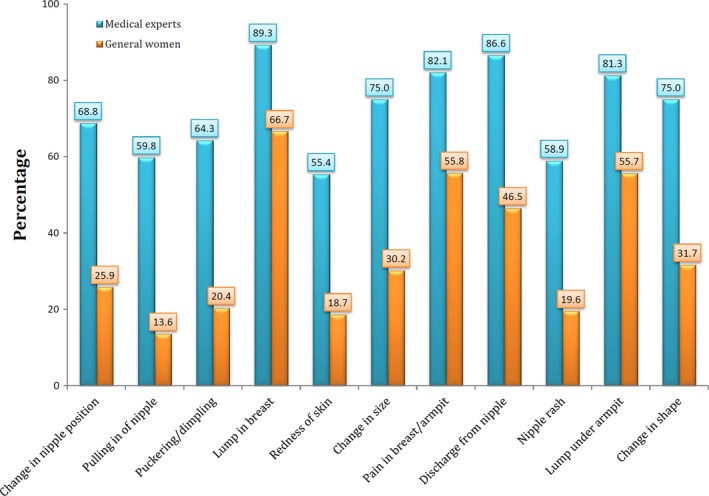
Distribution of correct knowledge about warning signs of breast cancer across two studied groups

**Figure 2:**
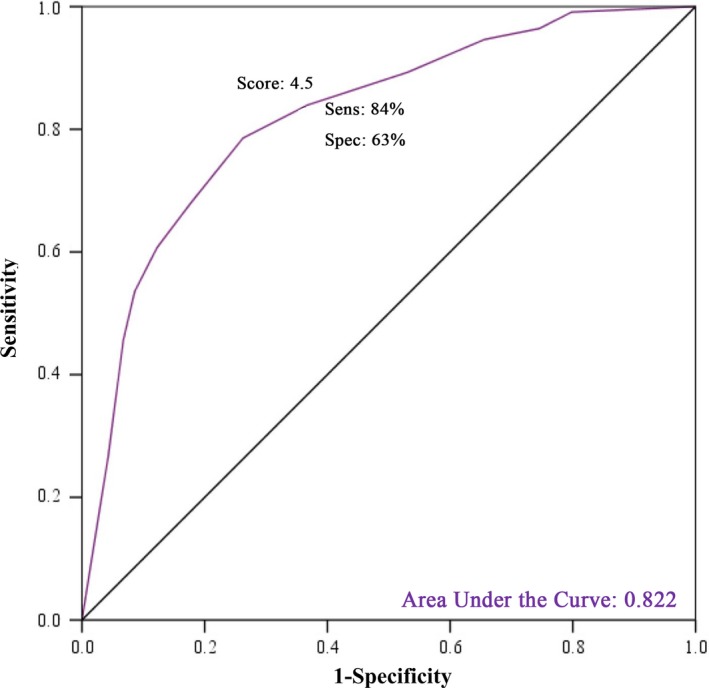
Ability of total score of awareness level about warning signs of breast cancer in BCAM for discriminating general women and medical/clinical expert by using ROC analysis

Table 3 provides the distribution of the correct answer to BCAM items between two studied groups (general women and medical/clinical experts). As can be seen, there were significant differences between two groups in terms of awareness about all breast cancer warning signs. As expected, awareness level about breast cancer warning signs was significantly higher in medical/clinical experts than general women (*P* < 0.0001; Table 3 and Figure 1). Discriminant validity of the BCAM subscale was evaluated based on total score of correct knowledge of all warning signs. Receiver operating characteristic (ROC) curve was generated (Area under the curve [AUC]) of 0.822 (95% CI: 0.781, 0.864); indicating strong accuracy for discriminating general women from medical/clinical experts with optimal cutoff point 4.5 (sensitivity, 84%, and specificity, 63%). Figure 2 demonstrates the ability of warning signs subscale of BCAM to discriminate between general women and medical/clinical experts. In addition, according to Table 4, it can be seen that the total score of warning signs subscale has significant discriminant validity and it is able to discriminate women with personal and family history of breast problems from general women (*P* = 0.001; Table 4).

**Table 3:** Distribution of correct answer to the BCAM items and its total score in studied groups

		General women (n = 965)	Medical/clinical experts (n = 113)	*P*‐value
Knowledge of breast cancer symptoms signs (yes)		722 (75.5)	110 (98.2)	<0.0001
Know if the following are warning signs (yes)	Change in nipple position	237 (25.9)	77 (68.8)	<0.0001
	Pulling in of nipple	124 (13.6)	67 (59.8)	<0.0001
	Puckering/dimpling	186 (20.4)	72 (64.3)	<0.0001
	Lump in breast	611 (66.7)	100 (89.3)	<0.0001
	Redness of skin	171 (18.7)	62 (55.4)	<0.0001
	Change in size	277 (30.2)	84 (75.0)	<0.0001
	Pain in breast/armpit	511 (55.8)	92 (82.1)	<0.0001
	Discharge from nipple	426 (46.5)	97 (86.6)	<0.0001
	Nipple rash	179 (19.6)	66 (58.9)	<0.0001
	Lump under armpit	510 (55.7)	91 (81.3)	<0.0001
	Change in shape	290 (31.7)	84 (75.0)	<0.0001
Total score of correct knowledge about all breast cancer warning signs		3.85 (3.02)	7.96 (3.00)	<0.0001
Knowledge of age‐related risk	A 30 y old	202 (21.4)	25 (22.3)	<0.0001
	50 y old	164 (17.4)	30 (26.8)	
	70 y old	20 (2.1)	4 (3.6)	
	A woman of any age	556 (59.0)	53 (47.3)	
Breast checking	Once a week	42 (4.4)	9 (8.0)	<0.0001
	Once a month	218 (22.9)	51 (45.1)	
	At least every 6 mo	235 (24.7)	24 (21.2)	
	Rarely or never	458 (48.1)	29 (25.7)	
Sources of gaining awareness (yes)	Radio	86 (8.9)	7 (6.2)	0.328
	TV	385 (40.0)	31 (27.4)	0.010
	Newspaper	254 (26.4)	30 (26.5)	0.98
	Friends	244 (25.3)	9 (8.0)	<0.0001
	Family	235 (24.4)	4 (3.5)	<0.0001
	Physician/Midwife	436 (45.3)	64 (56.6)	0.022
	Other sources	104 (10.8)	45 (39.8)	<0.0001

Values are mean (SD) or number (percent). *P*‐values are based on independent Student's *t* test or chi‐square test for continuous and categorical variables, respectively.

**Table 4:** Comparison of correct answer to the BCAM items and its total score between women with personal and family history of breast problems and general women

Warning signs	General women without family and personal history of breast problems (n = 666)		General women with family and personal history of breast problems (n = 297)		*P*‐value
	Yes	No	Yes	No	
Change in nipple position	142 (22.7)	484 (77.3)	95 (33.1)	192 (66.9)	0.001
Pulling in of nipple	71 (11.3)	555 (88.7)	53 (18.5)	234 (81.5)	0.004
Puckering/dimpling	113 (18.1)	511 (81.9)	73 (25.3)	215 (74.7)	0.012
Lump in breast	400 (63.9)	226 (36.1)	210 (72.9)	78 (27.1)	0.007
Redness of skin	108 (17.3)	518 (82.7)	63 (21.9)	225 (78.1)	0.096
Change in size	176 (28.1)	450 (71.9)	100 (34.7)	188 (65.3)	0.043
Pain in breast/armpit	330 (52.8)	295 (47.2)	181 (62.8)	107 (37.2)	0.004
Discharge from nipple	291 (46.5)	335 (53.5)	154 (53.5)	134 (46.5)	0.991
Nipple rash	111 (17.8)	513 (82.2)	68 (23.7)	219 (76.3)	0.037
Lump under armpit	332 (53.0)	294 (47.0)	177 (61.5)	111 (38.5)	0.017
Change in shape	196 (31.4)	429 (68.6)	93 (32.3)	195 (67.7)	0.779
Total score of correct knowledge about all breast cancer warning signs	3.63 (2.98)		4.36 (3.05)		0.001
Area under the curve [AUC] (95% CI)^a^	0.569 (0.530, 0.609)				

Values are mean (SD) or number (percent). *P*‐values are based on independent Student's *t* test or chi‐square test for continuous and categorical variables, respectively.

AUC based on the total score of awareness about all warning signs of breast cancer.

### 3.4 Construct validity

**Figure 3:**
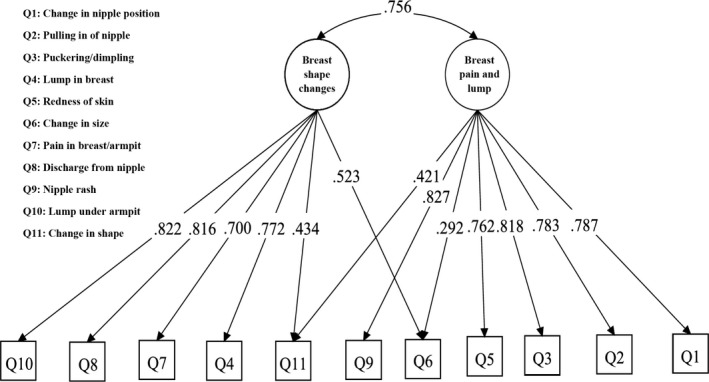
Confirmatory factor analysis testing the extracted construct from EFA on the BCAM items

Construct validity was evaluated using factor analysis and latent class analysis (LCA). EFA with Varimax rotation extracted two factors from the warning signs subscale of BCAM which were labeled as “breast shape changes” and “breast pain and lump” accounting for 26.98% and 23.67% of total variance, respectively. A KMO value of 0.896 and *P* < 0.05 for the Bartlett's test confirmed the data viability for factorability. Table 5 provides the factor loadings of two extracted factors from EFA on the 11 items of BCAM. The confirmatory factor analysis confirmed the adequacy of extracted construct from EFA, as shown in Figure 3. Values of goodness of fit indices were within predefined acceptable limits (Chi‐square/*df* = 2.9, RMSEA = 0.046; CFI = 0.984; TLI = 0.978); also all items loaded significantly on their respective factors (Figure 3).

**Table 5:** Factor loadings, class‐specific levels of correct awareness (%) about breast cancer's warning signs, the size of classes and item‐scale correlations

Warning signs	Exploratory factor analysis		Latent class analysis						Item‐scale correlations^a^
	Factor loadings		Class 1 (high awareness)		Class 2 (moderate awareness)		Class 3 (poor awareness)		
	Breast shape changes	Breast pain and lump	Yes	No	Yes	No	Yes	No	
Change in nipple position	0.645		83.19	16.81	25.06	74.94	0.25	99.75	0.472
Pulling in of nipple	0.731		63.7	36.3	8.82	91.18	0.04	99.96	0.462
Puckering/dimpling	0.718		81.9	18.1	15.17	84.83	2.45	97.55	0.502
Lump in breast		0.787	95.16	4.84	83.01	16.99	15.26	84.74	0.541
Redness of skin	0.635		69.26	30.74	15.78	84.22	0.06	99.94	0.450
Change in size	0.420	0.503	88.88	11.12	30.82	69.18	0.49	99.51	0.483
Pain in breast/armpit		0.686	90.45	9.55	68.68	31.32	9.65	90.35	0.606
Discharge from nipple		0.574	94.53	5.47	56.75	43.25	0.08	99.92	0.569
Nipple rash	0.691		82.36	17.64	14.84	85.16	0.05	99.95	0.522
Lump under armpit		0.751	95.11	4.89	71.18	28.82	0.31	99.69	0.590
Change in shape	0.507	0.464	94.02	5.98	32.08	67.92	0.5	99.5	0.499
Variance explained^b^ (%) and class size^c^ (%)	26.98%^b^	23.67%^b^	12.83%^c^		60.97%^c^		26.2%^c^		

Factor loadings <0.4 are not shown for simplicity.

a Phi's correlation coefficients between each item and its own domain (extracted classes) corrected for overlap.

b Variance explained resulted from factor analysis.

c Class size resulted from latent class analysis.

**Figure 4:**
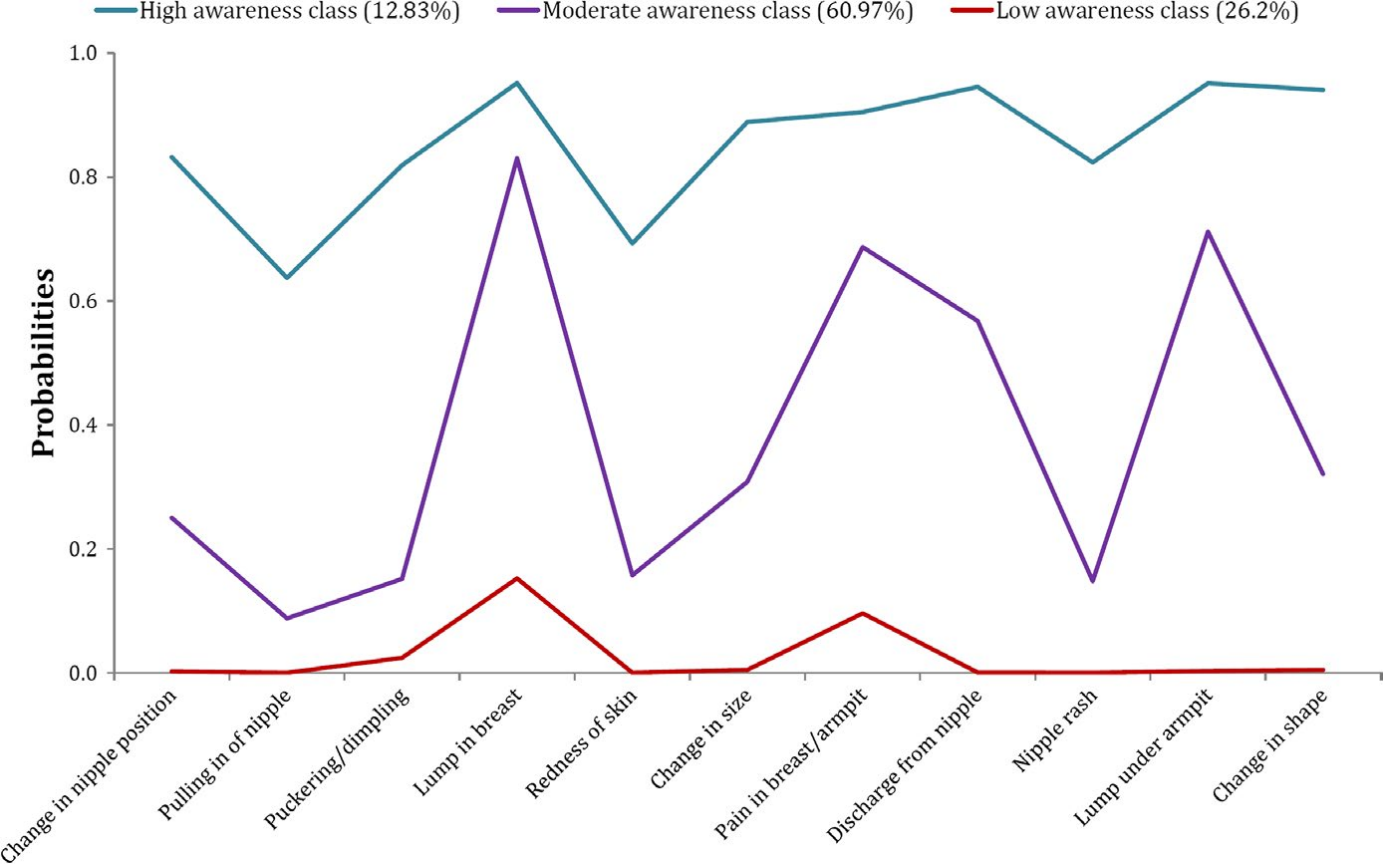
Graphical representation of class‐specific probabilities of correct knowledge about warning signs of breast cancer for three latent classes of study participants

Results of LCA on the 11 items of the warning signs subscale showed that model with three classes has highest quality of fit to the data (BIC = 9481.82, AIC = 9313.47). The entropy was 0.88 suggesting that individuals are correctly classified by our fitted model. Table 5 and Figure 4 show the prevalence of the correct answers to the questions about breast cancer warning signs in constructed classes. The nature of each class can easily be interpreted in terms of awareness levels about the warning signs. Accordingly, class 1 contains 12.83% of the study population and included general women with high awareness level and class 3, including 26.2% of participants, consisted of women with low awareness level. The second class included participants with mixed situation in terms of awareness levels about breast cancer warning signs (Table 5 and Figure 4). Then, we performed a split half cross‐validation, and performed an LCA on the first half (n = 455), followed by a CLCA on the second sample (n = 452). The LCA showed a three‐class solution which it was confirmed in the second half sample (BIC = 4766.9, AIC = 4622.9, and the entropy index = 0.89). Then, we repeated this process and used the second half sample as the training data. In this stage also the three extracted classes (BIC = 5021.85, AIC = 4877.6, and the entropy index = 0.88) were confirmed based on applying CLCA on the first half sample that it was considered as the validation sample. During this cross‐validation process, it was concluded that in average, 13% of participants were assigned to first class, 61% to second class, and 26% to third class.

### 3.5 The determinants of awareness levels about breast cancer warning signs

Table 6 presents the relation of some potential determinants of awareness levels about breast cancer warning signs in terms of latent class regression (LCR) coefficients. In the LCR model, the constructed classes considered as categories of dependent variable and the independent variables coefficients can be interpreted as odds ratios of belonging an individual to a certain class relative to reference class. Here, the third class (ie, poor awareness class) was considered as a reference. The independent variables coefficients in the first class show that the older age (OR = 1.06, 95% CI: (1.02, 1.09)), higher education level (collegiate category; OR = 23.96, 95% CI: (4.84, 118.58), and diploma category; OR = 13.83, 95% CI: (3.07, 62.19)), being married (OR = 2.64, 95% CI: (1.18, 5.91)) and having family history of breast cancer (OR = 2.15, 95% CI: (1.09, 4.26)) are significant determinates of high awareness levels. As can be seen from Table 6, job status and personal history of breast problems did not have significant impact on distinguishing between classes.

**Table 6:** Multivariable odds ratio (OR) and 95% confidence interval (95% CI OR) for the association of potential determinants of awareness levels about warning signs of breast cancer

Independent variables		Coefficients (SE)	OR (95% CI OR)	*z*‐Value (*P* value)
Class 2 (ref = Class 3)	Age	0.007 (0.01)	1.01 (0.99, 1.03)	0.71 (0.48)
	Educational level^a^			
	Collegiate (>12 y)	0.89 (0.27)	2.44 (1.43, 4.16)	3.28 (0.001)
	Diploma (12 y formal education)	0.46 (0.25)	1.59 (0.97, 2.59)	1.86 (0.06)
	Marital status^b^			
	Widow	0.77 (0.48)	2.15 (0.83, 5.54)	1.59 (0.11)
	Married	0.67 (0.26)	1.96 (1.19, 3.22)	2.63 (0.01)
	Job^c^			
	Retired	−0.46 (0.91)	0.63 (0.11, 3.75)	−0.51 (0.61)
	Student	0.12 (0.36)	1.13 (0.56, 2.27)	0.34 (0.73)
	Employee	−0.39 (0.24)	0.68 (0.42, 1.09)	−1.61 (0.11)
	Personal history (yes)	−0.091 (0.24)	0.91 (0.57, 1.46)	−0.38 (0.70)
	Family history (yes)	0.87 (0.25)	2.40 (1.46, 3.94)	3.44 (0.0006)
Class 1 (ref = Class 3)	Age	0.06 (0.02)	1.06 (1.02, 1.09)	3.46 (0.0005)
	Educational level^a^			
	Collegiate (>12 y)	3.18 (0.82)	23.96 (4.84, 118.58)	3.89 (0.0001)
	Diploma (12 y formal education)	2.63 (0.77)	13.83 (3.07, 62.19)	3.42 (0.0006)
	Marital status^b^			
	Widow	1.38 (0.77)	3.97 (0.87, 18.11)	1.78 (0.08)
	Married	0.97 (0.41)	2.64 (1.18, 5.91)	2.35 (0.02)
	Job^c^			
	Retired	−0.15 (1.05)	0.86 (0.11, 6.75)	−0.14 (0.89)
	Student	−0.50 (0.88)	0.61 (0.11, 3.42)	−0.57 (0.57)
	Employee	0.54 (0.33)	1.72 (0.90, 3.29)	1.63 (0.10)
	Personal history (yes)	−0.12 (0.39)	0.89 (0.41, 1.91)	−0.30 (0.76)
	Family history (yes)	0.77 (0.35)	2.15 (1.09, 4.26)	2.20 (0.03)

a Reference category is “Under Diploma and illiterate”.

b Reference category is “single”.

c Reference category is “housewife”.

### 3.6 Convergent validity

All item‐scale correlations based on phi's coefficients exceeded the set value of 0.40, indicating satisfactory convergent validity (Table 5). Item 7 (pain in breast/armpit) and item 10 (lump under armpit) showed the highest item‐scale correlation. In addition, item‐scale correlations based on eta's coefficients were between 0.45 and 0.58, indicating satisfactory item convergent validity.

### 3.7 Floor and ceiling effects

Ten percent of the participants achieved the minimum score of 0, and 14% of the respondents reached the maximum score of 11. These percentages were below the cutoff point of 15%, suggesting no floor and ceiling effect.

## 4 DISCUSSION

In current study, the psychometric properties (test‐retest reliability, internal consistency, discriminant validity, and construct validity) of the Persian version of BCAM were evaluated. To the best of our knowledge, this questionnaire, which is self‐report and quick to complete, is the first fully validated instrument to assess knowledge of breast cancer symptoms and age‐related risk and reported frequency of breast checking in Iranian women. The results showed that the Persian version of BCAM has excellent test‐retest reliability and internal consistency. Medical/clinical experts had higher levels of breast cancer awareness than general women and patients than general women, indicating good discriminant validity. Applying latent class analysis for evaluating of construct validity led to three classes (high, moderate, and low) in terms of awareness levels about early warning signs of breast cancer. The instrument showed satisfactory convergent validity, because all item‐scale correlation coefficients exceeded the cut‐off 0.40. No ceiling and floor effects were found. The main determinants of awareness level about early warning signs of breast cancer were educational level, marital status, family history of breast cancer, and age.

In the present study, the ICC index, as a measure of test‐retest reliability, for the total score of warning signs subscale of the BCAM was 0.84 which is acceptable. Previously reported ICC for the total score of signs and symptoms subscale of Thai version was 0.786.^9^ Regarding internal consistency, the warning signs subscale showed Cronbach's alpha coefficient of 0.882; suggesting high internal consistently. Previous studies had also reported Cronbach's alpha values of 0.890, and 0.745 in Arabic and Thai versions, respectively,^12^, ^26^ and 0.79 in Indonesian version.^8^ The test‐retest reliability of all items in Persian Version of the BCAM varied 0.47‐0.81 which was superior to those reported for the original questionnaire (0.28‐0.68),^7^ except for puckering/dimpling symptom. In line with Linsell et al's^7^ study, test‐retest reliability of the Persian version of BCAM was moderate to very good and the percentages agreement between the two measures were high for each item.

The SEM as a measure of absolute reliability, and the SDC, as a real change greater than the measurement error, were not evaluated for the original English version^7^ and for the other translated versions of BCAM^8^, ^12^, ^18^, ^26^; however, the estimated values of SEM and SDC were satisfactory in the current study, which indicates the reliability and responsiveness of the BCAM‐Persian. Accordingly, these indices (SDC and SEM) will be useful in interventional studies for evaluating the effectiveness of intervention.

According to our results, the Persian version of BCAM well discriminated general women and medical/clinical experts; in which knowledge level about warning signs, age‐related risk and breast checking were all significantly higher in the medical/clinical experts. This result indicated acceptable discriminant validity for this instrument. The result of Linsell et al's^7^ study was similar to our study except for “pain in breast/armpit,” “lump in breast,” and “lump under armpit” symptoms which did not differ between two studied groups in the aforesaid study.

In the current study, the evaluation of construct validity BCAM‐Persian by EFA led to extraction of two factors as “breast shape changes” and “breast pain and lump,” and the adequacy of extracted construct confirmed by CFA. The construct validity based on factor analysis was not evaluated in the original English version^7^; however, the results of other translated versions of BCAM partly were comparable with our findings.^8^, ^9^, ^12^, ^18^ They extracted one factor for the signs and symptoms domain.^18^ It is necessary to mention that the number of used items was different from our study, for example, Wachira et al used only the three items including “change in size of breast,” “change in size of nipple,” and “change in shape of breast and nipple.” For construct validity of the Persian version of BCAM, we also used an advanced statistical method, that is, LCA model that was more relevant based on the scale of the BCAM items. In this regard, our findings were not comparable to those reported in the original version and other studies^7^ that did not use this method. According to the results of LCA method, we observed that studied population could be classified into three latent classes in terms of awareness levels about breast cancer warning signs; the first class consisted of women with high levels of awareness (12.83%). In line with some previous studies,^6^, ^27^, ^28^ our results revealed that the majority of participants (87.17%) had moderate or inadequate knowledge about early warning signs of breast cancer that can be attributed to specific sociocultural characteristics of studied population.

In the current study, the most effective determinants of awareness levels about early warning signs of breast cancer were educational level, marital status, family history of breast cancer, and age, respectively. The highlighted role of educational status on awareness level in the present study was in agreement with previous studies.^6^, ^28^, ^29^, ^30^, ^31^, ^32^, ^33^, ^34^, ^35^, ^36^, ^37^ In addition, our findings in line with other studies showed a positive association between family history of breast cancer and high awareness levels about early warning signs^6^, ^29^, ^36^, ^37^, ^38^; however, in our study and few previous studies, the significant association was not observed between personal history of breast problems and knowledge levels about breast cancer.^39^ It is expected that the women with higher educational attainments and/or with a family or personal history of breast cancer were particularly likely to know the early warning signs of breast cancer.^40^ The current study showed significant association between age and knowledge levels about breast cancer early warning signs, also results indicated that married women had higher knowledge about warning signs of breast cancer. In these regards, there were similar findings with our results in some previous studies.^6^, ^37^, ^41^, ^42^, ^43^, ^44^, ^45^ It seems that older and married women are generally more concerned about their health, so they have more tendencies to get more information about BC determinants.

## 5 STUDY STRENGTHS AND LIMITATIONS

It is important to recognize some strengths and limitations of the current study. In contrast to the original questionnaire, which is only had been validated in older women (67‐73 years), our instrument was developed among a general women population (older than 18 years old). In addition, this study was carried out on a relatively large sample of the general women in the Isfahan city which is one of the cities located in central of Iran with high incidence rate of breast cancer.^46^ We used an advanced statistical method, that is, latent class analysis (LCA) to evaluate the construct validity of the Persian version of BCAM questionnaire. Despite these strengths, this study is not without limitations. We included women only from Isfahan (in the central of Iran); therefore, the representativeness of this sample for all Iranian women or other Persian language countries is not known. This instrument does not evaluate knowledge level of risk factors of breast cancer or health behavior related to breast cancer awareness. Finally, in the backward translation process of questionnaire, we only included one translator; however, according to the guidelines recommended by Beaton et al, it was better to include two translators.

## 6 CONCLUSIONS

In conclusion, the Persian version of BCAM questionnaire showed good psychometric properties in terms of test‐retest reliability, internal consistency, convergent, and discriminant validity, suggesting it will have utility in assessing breast cancer awareness about early warning signs in Persian language women. It is a self‐report questionnaire, inexpensive, and quick to complete for monitoring levels of breast cancer awareness, and evaluating the impacts of interventions attempting to raise breast cancer awareness. The results of the present study also indicated that studied population has a three‐class latent structure (high, moderate, and low awareness classes) in terms of knowledge levels about early warning signs of breast cancer more predominant by moderate and particularly low levels. The women with higher educational attainments, and family history of breast cancer, had higher levels of awareness, emphasizing on the need for raising awareness about breast cancer among Iranian women as an effective way to overcome increasing trend and burden of breast cancer in the population.

## CONFLICT OF INTEREST

The authors declare that they have no conflict of interest.
